# Correction: FGF2-FGFR1 signaling regulates release of leukemia-protective exosomes from bone marrow stromal cells

**DOI:** 10.7554/eLife.47174

**Published:** 2019-03-29

**Authors:** Nathalie Javidi-Sharifi, Jacqueline Martinez, Isabel English, Sunil K Joshi, Renata Scopim-Ribeiro, Shelton K Viola, David K Edwards, Anupriya Agarwal, Claudia Lopez, Danielle Jorgens, Jeffrey W Tyner, Brian J Druker, Elie Traer

Javidi-Sharifi N, Martinez J, English I, Joshi SK, Scopim-Ribeiro R, Viola SK, Edwards D, Agarwal A, Lopez C, Jorgens D, Tyner JW, Druker BJ, Traer E. 2019. FGF2-FGFR1 signaling regulates release of leukemia-protective exosomes from bone marrow stromal cells. *eLife* **8**:e40033. doi: 10.7554/eLife.40033.

Published 5, February 2019

The author list was updated to include SK Viola, who assisted with preparation of Figure 2 and optimized culturing of primary marrow stroma in the lab. He was inadvertently left of the original manuscript submission. R Scopim-Ribeiro’s name was also originally misspelled and is now corrected.

The article has been corrected accordingly.

