# Supplementary material for: FGF2-FGFR1 signaling regulates release of Leukemia-Protective exosomes from bone marrow stromal cells
Source: eLife. 2019 Feb 5;8:e40033. doi: 10.7554/eLife.40033 (PMC6363389; doi:10.7554/eLife.40033)
Supplement: Supplementary file 1. [file elife-40033-supp1.docx]

**Supplemental Table**

Table 1. Information on all immunoblot antibodies used in the paper and supplements.

| **Target** | **Antibody Product #** | **Vendor** | **Clone** | **Species** | **Primary Dilution** | **Secondary Antibody** | **Secondary Dilution** | **Predicted Size (kDa)** |
| --- | --- | --- | --- | --- | --- | --- | --- | --- |
| FGFR1 | 9740 | Cell Signaling | D8E4 | Rb | 1:1000 | LiCor IRDye 800CW Goat anti-Rabbit IgG (Product #: 926-32211) | 1:15,000 | 92, 120, 145 |
| FGF2 | Sc-79 | Santa Cruz |  | Rb | 1:500 | LiCor IRDye 800CW Goat anti-Rabbit IgG (Product #: 926-32211) | 1:15,000 | 20 |
| CD63 | ab134045 | ABCAM | EPR5702 | Rb | 1:1000 | LiCor IRDye 800CW Goat anti-Rabbit IgG (Product #: 926-32211) | 1:15,000 | 63 |
| CD9 | Sc-9148 | Santa Cruz | H-110 | Ms | 1:200 | LiCor IRDye 680RD Goat anti-Mouse IgG (Product #: 926-68070) | 1:15,000 | 21-30 |
| Tsg-101 | Sc-7964 | Santa Cruz |  | Ms | 1:200 | LiCor IRDye 680RD Goat anti-Mouse IgG (Product #: 926-68070) | 1:15,000 | 45 |
| Actin | MAB1501 | Millipore | C4 | Ms | 1:5000 | LiCor IRDye 680RD Goat anti-Mouse IgG (Product #: 926-68070) | 1:15,000 | 43 |
